# SARS-CoV-2 Seroprevalence in a University Community: A Longitudinal Study of the Impact of Student Return to Campus on Infection Risk Among Community Members

**DOI:** 10.1101/2021.02.17.21251942

**Published:** 2021-02-26

**Authors:** Callum R.K. Arnold, Sreenidhi Srinivasan, Catherine M. Herzog, Abhinay Gontu, Nita Bharti, Meg Small, Connie J. Rogers, Margeaux M. Schade, Suresh V Kuchipudi, Vivek Kapur, Andrew Read, Matthew J. Ferrari

**Affiliations:** 1 Department of Biology, Pennsylvania State University, University Park, PA, USA 16802; 2 Center for Infectious Disease Dynamics, Pennsylvania State University, University Park, PA, USA 16802; 3 Huck Institutes of the Life Sciences, Pennsylvania State University, University Park, PA, USA 16802; 4 Department of Veterinary and Biomedical Sciences, Pennsylvania State University, University Park, PA, USA 16802; 5 College of Health and Human Development, Pennsylvania State University, University Park, PA, USA 16802; 6 Social Science Research Institute, Pennsylvania State University, University Park, PA, USA 16802; 7 Department of Nutritional Sciences, Pennsylvania State University, University Park, PA, USA 16802; 8 Department of Animal Science, Pennsylvania State University, University Park, PA, USA 16802

## Abstract

**Importance:**

The Coronavirus Disease 2019 (COVID-19) pandemic has had wide-spread impacts on health and behavioral patterns. Returning university students represent large-scale, transient demographic shifts and a potential source of transmission risk to adjacent communities.

**Objective:**

Test the *a priori* hypothesis that the Fall 2020 student return-to-campus would correlate with an increase in infection rates in the surrounding community; identify risk factors for SARS-CoV-2 infection within and between the subgroups.

**Design:**

Prospective longitudinal cohort study.

**Setting:**

Conducted in Centre County, Pennsylvania, USA from August 2020 to December 2020.

**Participants:**

Non-random cohorts of county residents 18 years of age or older living in Centre County prior to the return to in-person instruction at the Pennsylvania State University and returning students who were enrolled during the Fall 2020 term.

**Exposure(s):**

Contact with known SARS-CoV-2-positive and COVID-symptomatic individuals, attending gatherings of various sizes, and adherence to public health non-pharmaceutical interventions.

**Main Outcome(s) and Measure(s):**

Presence of IgG antibodies against the SARS-CoV-2 surface spike glycoprotein (S) receptor-binding domain (S/RBD), measured using an indirect isotype-specific (IgG) screening enzyme immunoassay.

**Results:**

Of 345 community participants, 19 (5.5%) were positive for SARS-CoV-2 IgG antibodies at their first visit between 7 August and 2 October. Of 625 returning student participants, 195 (31.2%) were positive for SARS-CoV-2 antibodies between 26 October and 23 November. 28 (8.1%) of the community participants had returned a positive result by 9 December. Only contact with known SARS-CoV-2-positive individuals and attendance at small gatherings (20–50 individuals) were significant predictors of IgG antibodies among returning students (aOR, 95% CI: 3.24, 2.14–4.91, p<0.001; 1.62, 1.08–2.44, p<0.05; respectively).

**Conclusions and Relevance:**

Despite high seroprevalence observed within the student population, seroprevalence in a longitudinal cohort of community residents was low and stable from before student arrival for the Fall 2020 term to after student departure, implying limited transmission between these cohorts. This implies the potential to minimize the transmission of SARS-CoV-2 from high prevalence, geographically coincident sub-populations. Future work should investigate the relative efficacy of specific measures to reduce risk of transmission from itinerant to resident populations and guide communities with high levels of episodic migration and similar social configurations.

## BACKGROUND

Demographic shifts, high population densities, and population mobility are known to impact the spread of infectious diseases.^1–5^ While this has been well characterized at large scales^6–8^, it has proved more challenging to demonstrate at smaller geographic scales.^9–11^ In the context of the COVID-19 pandemic, the return of college and university students to in-person and hybrid (in-person and online) instruction in the Fall 2020 term represented a massive demographic shift in many communities in the United States (US); specifically, increased total population and proportion living in high density living facilities, with a concomitant increase in person-to-person interactions.^12^ This shift had the potential to increase SARS-CoV-2 transmission within returning student populations and to surrounding community resident populations, particularly for non-urban campuses where incidence lagged behind larger population centers.^13^ Modeling analyses conducted prior to student return raised concerns that university re-opening would result in significant SARS-CoV-2 transmission in both the returning student and community resident populations.^14,15^

During the Fall 2020 term, many universities in the US experienced high rates of COVID-19 cases among students,^16^ with a 56% increase in incidence among counties home to large colleges or universities relative to matched counties without such institutions.^12^ While there is strong evidence of high incidence rates associated with a return to campus at US colleges and universities,^12^ the increase in risk in surrounding communities, and the rate of transmission from campuses to communities, have been less well characterized.

This investigation reports the results of a longitudinal serosurvey of community residents in Centre County, Pennsylvania, USA, which is home to The Pennsylvania State University (PSU), University Park (UP) campus. The return of approximately 35,000 students to the UP campus in August 2020 represented a nearly 20% increase in the county population size.^17^ During the Fall 2020 term, more than 4,500 cases of SARS-CoV-2 infections were detected among the student population.^18^ Between 7 August and 2 October 2020 (before and just after student return), we enrolled a cohort of community residents and tested serum for the presence of anti-Spike Receptor Binding Domain (S/RBD) IgG, which would indicate prior SARS-CoV-2 exposure.^19^ This was repeated in the same cohort during December 2020 (post-departure of students), and we present seroprevalence for both sampling waves. Additionally, returning students were enrolled in a longitudinal cohort, and IgG seroprevalence results are presented from the first wave of sampling (between October and November 2020, prior to the end of the term). The hypothesis tested was that the rapid influx of students during the Fall 2020 term would be correlated with increased prevalence of SARS-CoV-2 within the geographically co-incident community.

## METHODS

### Design, Setting, and Participants

This human subjects research was conducted with PSU Institutional Review Board approval and in accordance with the Declaration of Helsinki. The study uses a longitudinal cohort design, with two separate cohorts: community residents and returning students. We report on measures from the first two clinic visits for the community resident cohort and the first clinic visit for the returning student cohort.

To assist with recruitment into studies under the Data4Action (D4A) Centre County COVID Cohort Study umbrella, a REDCap survey was distributed to residents of Centre County, where respondents could indicate interest in participating in future studies and provide demographic data. Returning students received a similar survey to express interest in study participation and were also recruited through cold-emails and word-of-mouth.

Individuals were eligible for participation in the community resident cohort if they were: ≥18 years old, residing in Centre County at the time of recruitment (June through September 2020); expecting to reside in Centre County until June 2021; fluent in English; and capable of providing their own consent. PSU students who remained in Centre County through spring and summer university closure were eligible for inclusion in the community resident cohort as this group experienced similar geographic COVID-19 risks as community residents (versus returning students who experienced variable risk during spring and summer depending on their residence).

Participants were eligible for participation in the returning student cohort if they were: ≥18 years old; fluent in English; capable of providing their own consent; residing in Centre County at the time of recruitment (October 2020); officially enrolled as PSU UP students for the Fall 2020 term; and intended to be living in Centre County through April 2021.

In both cohorts, individuals were invited to participate in the survey-only portion of the study if they were: lactating, pregnant, or intended to become pregnant in the next 12 months; unable to wear a mask for the clinic visit; demonstrated acute COVID-19 symptoms within the previous 14 days; or reported a health condition that made them uncomfortable with participating in the clinic visit.

Upon enrollment, returning students were supplied with a REDCap survey to examine socio-behavioral phenomena, such as attendance at gatherings and adherence to non-pharmaceutical interventions, in addition to information pertaining to their travel history and contact with individuals who were known or suspected of being positive for SARS-CoV-2. Community residents received similar surveys at both time points with questions relating to potential SARS-CoV-2 household exposures. All eligible participants were scheduled for a clinical visit at each time interval where blood samples were collected for ELISA testing.

### Outcomes

The primary outcome was the presence of S/RBD IgG antibodies, measured using an indirect isotype-specific (IgG) screening ELISA developed at PSU.^20^ Further details in the Supplement. The presence of anti-SARS-CoV-2 antibodies has been documented in prior seroprevalence studies as a method of quantifying cumulative exposure.^21–23^

### Statistical Methods

Community resident and returning student cohorts’ seroprevalence are presented with binomial 95% confidence intervals. We estimated each subgroup’s true prevalence, accounting for imperfect sensitivity and specificity of the IgG assay, using the *truePrev()* function in the *prevalence* package in R. We calculated a 95% binomial confidence interval for test sensitivity of the IgG assay for detecting prior self-reported positive tests in the returning student cohort (students had high access to testing from a common University provider) and assumed a uniform prior distribution between these limits. Estimates of the true prevalence were then calculated across all possible values of specificity between 0·85 and 0·99. We did not estimate prevalence corrected for demographics as participants were not enrolled using a probability-based sample. We assessed demographic characteristics of the tested participants relative to all study participants to illustrate potential selection biases (Table 1).

For contingency tables and raw odds ratios, we used two-sided Fisher’s exact test to determine significance at alpha = 0·05 and present 95% confidence intervals. Pairwise Fisher’s exact test of proportions was used with the Bonferroni-Holm correction in the *r* × *c* case, and Welch’s two-sample t-test to compare distributions.

Missing values were deemed “Missing At Random” and imputed, as described in the Supplement. We estimated the adjusted odds ratios (aOR) of IgG positivity in the returning student subgroup using multivariable logistic regression implemented with the *mice* and *finalfit* packages. We considered the following risk factors: close proximity (6 feet or less) to an individual who tested positive for SARS-CoV-2; close proximity to an individual showing key COVID-19 symptoms (fever, cough, shortness of breath); attendance at a small gathering (20–50 people) in the past 3 months; attendance at a medium gathering (51–200 people) in the past 3 months; live in University housing; ate in a restaurant in the past 7 days; ate in a dining hall in the past 7 days; only ate in their room/apartment in the past 7 days; travelled in the 3 months prior to returning to campus; and travelled since returning to campus for the Fall term. Given the airborne transmission of SARS-CoV-2, all the above variables are potential exposure risks due to the increased contact with individuals outside of a participants’ household.^24–27^

All statistical analyses were conducted using R – version 4.03 (2020-10-10), with a pipeline created using the *targets* package.

## RESULTS

A total of 10,369 community residents were identified through an initial REDCap survey that collected eligibility, demographic, and contact information. Of those who identified willingness to participate in a later study, 1,540 were contacted and enrolled. 1,432 completed a first clinic visit between 7 August and 2 October 2020, and 345 of those completed a second clinic visit between 30 November and 9 December 2020 and for whom both visit 1 and visit 2 samples were analysed using the in-house ELISA assay; the remaining participants’ samples will be analysed in the future. 1,349 returning students were recruited using volunteer sampling and 707 enrolled; of these, 625 completed clinic visits for serum collection between 26 October and 23 November. 642 students indicated willingness to participate but did not complete intake procedures or did not attend their scheduled clinic visit.

Among participants with serum samples: the median age community residents was 47 years (IQR: 35·0–60·0), with 81·7% between the ages 18–65 years, and for the returning students the median age was 20·9 years (IQR: 19·7–21·6), with 100% between the ages 18–65 years; 62·0% of the community residents identified as female and 33·6% as male; 64·6% of the returning students identified as female and 34·4% as male; 90·1% of the community residents identified as white, as did 81·3% of the students. Similar proportions were seen in those enrolled without samples, and among the initial REDCap survey respondents (Table 1; Table 2).

**Figure 1:**
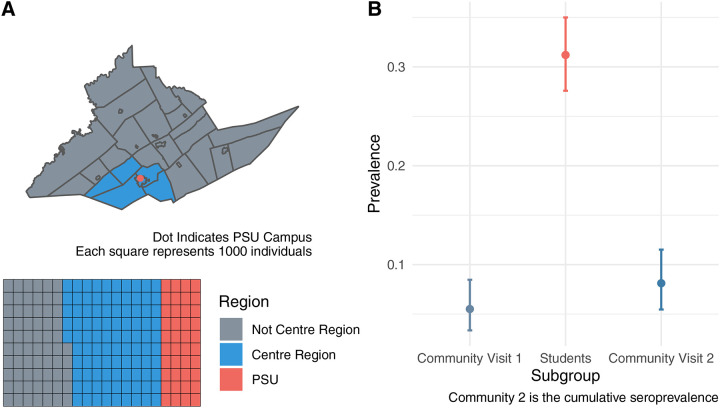
**A)** Map of Centre County, Pennsylvania, USA. Blue indicates the 5 townships and 1 borough that comprise the Centre Region. Red indicates the location of The Pennsylvania State University (PSU), University Park (UP) Campus. Inset illustrates the proportion of the county population in each region; PSU indicates the estimated student population that returned to campus for the Fall 2020 term. **B)** Raw seroprevalence (circles) with 95% binomial confidence intervals for the community residents at the first visit at the start of the Fall 2020 term (light blue), returning students at the end of the fall 2020 term (red), and community residents at either the first or the second visit after student departure (dark blue).

Although all county residents were eligible for participation, 77·0% of community resident participants were from the 5 townships (College, Ferguson, Harris, Half Moon, Patton) and 1 borough (State College) that form the “Centre Region” and account for ~59% of Centre County’s population^17^ (Figure 1). The median household income group in the community residents providing samples was $100,000–149,999 USD (IQR: $50,000–74,999; $100,000–149,999). The median household income in the county is $60,403.^17^ 47.4% of the county is female, 87·9% white, and 70·3% are between the ages of 18–65 years old.^17^ The study cohort is moderately older and more affluent (in part because of the exclusion of returning students), and disproportionately female compared to the general Centre County population.

Of the returning student participants, 665 (94·1%) had at least one test prior to enrollment in the study; of these, 105 (15·8%) self-reported a positive result (Table S3). Of these, 98 (93·3%) indicated that this test result occurred after their return to campus (median: 26 September; IQR: 13 September, 9 October). Of the 625 returning students with an ELISA result, 84 of the 90 (93·3%) with a self-reported prior positive test result were positive for SARS-CoV-2 IgG antibodies. Of the 535 returning students with ELISA results who did not report a positive SARS-CoV-2 test, 111 (20·7%) were positive for SARS-CoV-2 IgG antibodies. Of the total 625 returning students with ELISA results, 195 (31·2%) were positive for SARS-CoV-2 IgG antibodies (Figure 1).

Among the community resident participants, 19 of 345 (5·5%) were positive for SARS-CoV-2 antibodies at their first visit (Figure 1). Between their first and second visit, 9 participants converted from negative to positive and 9 converted from positive to negative; 28 (8·2%) were positive for SARS-CoV-2 IgG antibodies at either visit (Figure 1).

**Figure 2:**
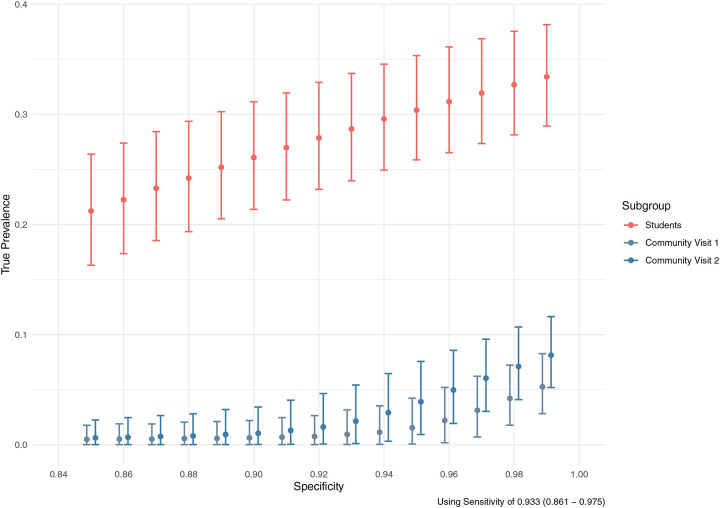
Estimated true prevalence (circles, with 95% confidence intervals) among participants at each sampling interval corrected for estimated assay sensitivity as a function of the assumed assay specificity (x-axis). Light blue indicates community residents at the first visit at the start of the Fall 2020 term, red indicates returning students at the end of the Fall 2020 term, and dark blue indicates community residents at the second visit after student departure.

To estimate true prevalence of SARS-CoV-2 exposure in our cohorts, the seroprevalence was corrected for the sensitivity and specificity of the assay. 93·3% (95% CI: 86–97%) of returning students with a self-reported prior positive SARS-CoV-2 test had positive IgG antibodies; this was used as an estimate of sensitivity of the IgG assay for detecting previously detectable infection (see Supplement for an alternative calculation of sensitivity that includes community resident responses). For all assumed levels of specificity, the 95% credible intervals for the true prevalence in the community residents overlapped for the pre- and post-term time points, and neither overlapped the 95% credible interval for the true prevalence estimate in the returning student subgroup (Figure 2).

Among the returning student subgroup, only close proximity to a known SARS-CoV-2-positive individual and attending small gatherings in the past 3 months (20–50 individuals) were significantly associated with a positive ELISA classification at alpha = 0·05 (aOR: 3·24, 2·14–4·91, p<0·001; aOR: 1·62, 1·08–2·44, p<0·05; respectively) in the multivariable model (Table 3). Taken individually, attending small gatherings (20–50 people) (OR: 2·38, 1·66–3·43, p<0·001), attending medium gatherings (51–200 people) (OR:1·88, 1·19–2·94, p<0·01), and close proximity to a known SARS-CoV-2-positive or symptomatic individual (OR: 3·65, 2·51–5·37, p<0·001; 1·81, 1·24–2·64, p<0·01; respectively) were all associated with the IgG positivity in crude calculations of association. Given the low prevalence observed within the community subgroup, and lack of significant effects observed during crude analysis, models were not fit to the community resident data.

Both the returning student and community resident participants self-reported high compliance with masking; 86·4% and 76·9%, respectively, reported always wearing mask or cloth face covering when in public (Table S4, Table S5). By contrast, less than one third of both groups (28·8% and 31·0%, respectively) self-reported always maintaining 6-feet of distance from others in public. Less than half (42·9%) of returning students indicated that they always avoided groups of 25 or greater, in contrast with 65·7% of community residents.

## DISCUSSION

The return of students to in-person instruction on the PSU UP campus was associated with a large increase in COVID-19 incidence in the county, as evidenced by over 4,500 student cases at PSU.^18^ In a sample of 625 returning students, 31% were positive for SARS-CoV-2 antibodies. Out of approximately 35,000 students who returned to campus, this implies that the detected cases may account for ~40% of all infections among PSU UP students. Despite this high incidence of SARS-CoV-2 infection in the county during the Fall 2020 term, a cohort of community residents, who disproportionately identified as female and lived in close proximity to campus, saw only a modest increase in the prevalence of SARS-CoV-2 IgG antibodies (5·5 to 8·1%) between September and December 2020. The true prevalence of prior SARS-CoV-2 infection in the cohorts depends on the assumed sensitivity and specificity. However, for all realistic values of sensitivity and specificity, there was no evidence of a significant increase in true prevalence in the community resident sample. While in-person student instruction has been associated with an increase in per-capita COVID-19 incidence at the aggregate level,^12^ these results suggest that outbreaks in the returning student and the community resident cohort we studied were asynchronous, implying limited between-cohort transmission. A recent analysis of age-specific movement and transmission patterns in the US suggested that individuals between the ages of 20–34 disproportionately contributed to spread of SARS-CoV-2;^28^ however, despite close geographic proximity to a college-aged population, transmission in our community resident sample appears distinctly lagged; suggestive of the potential for health behaviours to prevent infection.

Within the student group, presence of SARS-CoV-2 antibodies was significantly associated with close proximity to known SARS-CoV-2-positive individuals and attendance of small events, however, no other risk factors were correlated with an increase in SARS-CoV-2 IgG test positivity, aligning with other research.^23^ However, it is not possible to discern how much the likelihood of contact with a SARS-CoV-2 positive individual is due to the high campus prevalence versus individual behaviours. To the extent that limiting gatherings may reduce risk for students, it is not clear to what extent that will affect transmission from students to community residents. Both community residents and returning students reported high adoption of masking (>75%) and low adoption of distancing in public (31·0% and 28·8%, respectively). Returning students reported lower adherence to avoiding gatherings of greater than 25 individuals than community residents.

Neither the resident nor the student participants were selected using a probability-based sample. Thus, these participants may not be representative of any particular population. Those who chose to participate in this study may have been more cognizant and compliant with public health mitigation measures than non-participants. Specifically, the resident participants disproportionately lived in the townships immediately surrounding the UP campus, where extensive messaging^29^ and preventative campaigns were enacted, and they have a higher median income than the residents of Centre County overall.

Though the participants reflect a convenience sample, the difference in SARS-CoV-2 seroprevalence suggest that the cohorts did not experience a synchronous, well-mixed epidemic despite their close geographic proximity. College campuses have been observed to have high COVID-19 attack rates, and counties containing colleges and universities have been observed to have significantly higher COVID-19 incidence than demographically matched counties without such institutions.^12^ Thus, while college and university operations may present a significant exposure risk (because of large numbers of returning students, high density housing, and frequent in-person socialization), this analysis suggests the possibility that local-scale heterogeneity in mixing may allow for asynchronous transmission dynamics despite close geographic proximity. Thus, the disproportionately high incidence in the student population, which comprises less than one quarter of the county population, may bias assessment of risk in the non-student resident population. Risk assessment in spatial units (e.g., counties) that have strong population sub-structuring should consider these heterogeneities and their consequences for policy.

SARS-CoV-2 transmission between the student and community resident populations is likely to have occurred (perhaps multiple times) and these results do not preclude that possibility. However, the large difference in seroprevalence between the student and resident participants after the Fall term are consistent with either rare transmission events between students and residents, non-persistent transmission with the community residents, or both. We note that this was achieved in the context of disproportionate investment in prevention education, testing, contact tracing, and infrastructure for isolation and quarantine by PSU in the high-prevalence sub-population (students). Continued research in these cohorts may help identify mechanisms that mitigate transmission from and within high density, mobile sub-populations (e.g., displaced populations, seasonal workers, military deployment).

With respect to the health behaviors measured, the community resident and returning student groups differed only in their small social group contact, and thus a next step is to identify factors that may explain this difference (e.g., differences in leisure time activity norms and/or perceptions of age-related risk; business closures; university policies and sanctions). Minimizing risk, however, may come at significant social, psychological, educational, economic, and societal costs.^30^ Thus, operational planning for both institutions of higher education and the communities in which they are embedded should consider both the risk of SARS-CoV-2 transmission and the costs of mitigation efforts.

## Supplementary Material

Supplement 1

## Figures and Tables

**Table 1:** Demographic characteristics of study participants. Non-D4A participants are all participants in the initial anonymous survey from which Data4Action participants were drawn. D4A participants are divided into subsets for which antibody assays were conducted (N=345) and those for which assays were not conducted (N=1195).

	D4A Participant (N=1540)		Not D4A Participant (N=8826)
Variable	Assay Subset (N=345)	Non-Assay Subset (N=1195)	
**Age**			
Median [IQR]	47·0 [35·0, 60·0]	46·0 [36·0, 58·0]	46·0 [33·0, 59·0]
Missing	1 (0·3%)	11 (0·9%)	335 (3·8%)
**Race**			
White	311 (90·1%)	1057 (88·5%)	6075 (68·8%)
Aggregated Category*	18 (5·2%)	84 (7·0%)	1289 (14·6%)
Listed more than one race or ethnicity	15 (4·3%)	41 (3·4%)	159 (1·8%)
Missing	1 (0·3%)	13 (1·1%)	1303 (14·8%)
**Gender**			
Female	214 (62·0%)	784 (65·6%)	0 (0%)
Male	116 (33·6%)	337 (28·2%)	0 (0%)
Non-binary / Transgender / Self-described	6 (1·7%)	8 (0·7%)	0 (0%)
Prefer not to answer	1 (0·3%)	1 (0·1%)	0 (0%)
Missing	8 (2·3%)	65 (5·4%)	8826 (100%)
**Household Income**			
$200,000 and over	28 (8·1%)	134 (11·2%)	677 (7·7%)
$150,000 to $199,999	49 (14·2%)	158 (13·2%)	767 (8·7%)
$100,000 to $149,999	91 (26·4%)	311 (26·0%)	1502 (17·0%)
$75,000 to $99,999	41 (11·9%)	171 (14·3%)	1091 (12·4%)
$50,000 to $74,999	47 (13·6%)	149 (12·5%)	963 (10·9%)
$25,000 to $49,999	46 (13·3%)	123 (10·3%)	1462 (16·6%)
Under $25,000	9 (2·6%)	44 (3·7%)	259 (2·9%)
Prefer not to answer	33 (9·6%)	93 (7·8%)	801 (9·1%)
Missing	1 (0·3%)	12 (1·0%)	1304 (14·8%)

* Asian; Hispanic, Lantino/a, or Spanish; Black or African American; Middle Eastern or North African; Native American or Alaska Native; other race or ethnicity. This category is aggregated to protect participant identities because no single group comprised >4% of participants.

**Table 2:** Demographic characteristics of the returning student participants.

Variable	PSU Subset (N=625)
**Age**	
Median [IQR]	20·8 [19·7, 21·6]
Missing	10 (1·6%)
**Race**	
White	508 (81·3%)
Aggregated Category*	81 (13·0%)
Listed more than one race	30 (4·8%)
Missing	6 (1·0%)
**Gender**	
Female	404 (64·6%)
Male	215 (34·4%)
Genderqueer/nonconforming/transgender/different identity	5 (0·8%)
Missing	1 (0·2%)
**University Housing**	
Not Uni housing	445 (71·2%)
Uni housing	178 (28·5%)
Missing	2 (0·3%)

* Asian; Black or African American; American Indian or Alaska Native; Native Hawaiian or other Pacific Islander; some other race. This category is aggregated to protect participant identities because no single group comprised >4% of participants.

**Table 3:** Crude and adjusted odds ratios (aOR) of risk factors among returning PSU UP student cohort

Variable/Response	No/total (%) with SARS-CoV-2 antibodies	Crude OR (95% CI)	MICE Multivariable OR (95% CI)
Close proximity to known COVID-19 Positive Individual			
No	57/316 (18·04)	1 [Reference]	1 [Reference]
Yes	137/307 (44·63)	3·65 (2·515·37)***	3·24 (2·144·91)***
Attended a gathering of 20–50 people since arrival for the Fall Semester			
No	75/332 (22·59)	1 [Reference]	1 [Reference]
Yes	119/290 (41·03)	2·38 (1·663·43)***	1·62 (1·08–2·44)*
Ate in a dining hall in the past 7 days			
No	150/500 (30·00)	1 [Reference]	1 [Reference]
Yes	43/121 (35·54)	1·29 (0·82–1·99)	1·40 (0·79–2·48)
Attended a gathering of 51–200 people since arrival for the Fall Semester			
No	146/510 (28·63)	1 [Reference]	1 [Reference]
Yes	46/107 (42·99)	1·88 (1·19–2·94) **	1·26 (0·77–2·06)
Travelled in the 3 months prior to campus arrival			
No	74/263 (28·13)	1 [Reference]	1 [Reference]
Yes	114/339 (33·63)	1·29 (0·90–1·87)	1·15 (0·78–1·70)
Ate in a restaurant in the past 7 days			
No	90/317(28·39)	1 [Reference]	1 [Reference]
Yes	102/305 (33·44)	1·27 (0·89–1·81)	1·10 (0·75–1·62)
Only ate in their room in the past 7 days			
No	70/214 (32·71)	1 [Reference]	1 [Reference]
Yes	123/409 (30·07)	0·88 (0·61–1·28)	0·97 (0·64–1·46)
Close proximity to individual showing COVID-19 symptoms			
No	121/442 (27·38)	1 [Reference]	1 [Reference]
Yes	73/180 (40·56)	1·81 (1·24–2·64)**	0·89 (0·59–1·36)
Travelled since campus arrival			
No	80/255 (31·37)	1 [Reference]	1 [Reference]
Yes	114/368 (30·98)	0·98 (0·69–1·41)	0·87 (0·59–1·27)
Lives in University housing			
No	140/445 (31·46)	1 [Reference]	1 [Reference]
Yes	54/178 (30·34)	0·95 (0·64–1·40)	0·82 (0·50–1·36)

* p < 0·05 (2-tailed)

** p < 0·01 (2-tailed)

*** p < 0·001 (2-tailed)

## KEY POINTS

### Question

What was the effect of a large population influx on SARS-CoV-2 seroprevalence in an immunologically naïve resident population?

### Findings

Despite high seroprevalence observed within the student population, seroprevalence in a longitudinal cohort of community residents remained low and stable from before student arrival for the Fall term to after their departure, implying limited transmission between these subpopulations. Contact with an individual known to be SARS-CoV-2 positive and attending small gatherings were the only significant risk factors among returning students.

### Meaning

The demographic shift associated with student return to campus was not associated with increased SARS-CoV-2 seroprevalence in this cohort of community residents.
