# Supplementary material for: SARS-CoV-2 Seroprevalence in a University Community: A Longitudinal Study of the Impact of Student Return to Campus on Infection Risk Among Community Members
Source: medRxiv. 2021 Feb 26:2021.02.17.21251942. Originally published 2021 Feb 19. Preprint. [Version 3] doi: 10.1101/2021.02.17.21251942 (PMC7899462; doi:10.1101/2021.02.17.21251942)
Supplement: Supplementary file 1 [file media-1.docx]

# Supplemental Appendix

## Supplementary Text 

## Laboratory Methods:

### Production and Purification of SARS-CoV-2 Receptor-binding domain (S/RBD)

Transfections of plasmid pSL1510 (pCAGGS-RBD from Florian Krammer, Mount Sinai, USA) was performed using the Expi293 Expression System from ThermoFisher. Cells were cultured per manufacturer’s instructions (37°C, 8% CO2, in shaker flasks at 120-130 rpm), and the supernatant was harvested by simple centrifugation on the third day for downstream processing. Cell viability and concentration were monitored throughout to ensure that the culture remained in log phase growth. The detailed protocol is deposited in protocols.io^1^. Briefly, culture supernatant was incubated with pre-equilibrated Ni-NTA resin in 1X PBS at 4°C for 1 h on a nutator, after which a gravity column was used to elute the protein. 

### Estimation of IgG antibodies against SARS-CoV-2

An in-house indirect isotype-specific (IgG) ELISA against SARS-CoV-2 receptor-binding domain (S/RBD) was developed^1^. Commercially purchased human monoclonal antibody reactive to spike regions of SARS-CoV-1 and SARS-CoV-2 were used as positive controls in the assay (Two isotypes of CR3022, IgG1: Ab01680-10.0; Absolute Antibody, USA). The cut-off for this IgG ELISA was determined as an optical density (absorbance at 450 nm) higher than three or six standard deviations above the mean of the tested pre-COVID-19 serum samples (n=100). Briefly, serum was separated from the blood collected from study participants and inactivated at 56°C for 30 minutes. Microtiter plates were coated with purified recombinant S/RBD. Negative serum control was included on each microtiter plate. 1:50 dilutions of serum were added, incubated for 1 hour, washed, incubated with goat anti-human IgG (Fc specific) (A0170, Sigma-Aldrich, USA), and washed. 3,3′,5,5′-Tetramethylbenzidine dihydrochloride (TMB) was used as the ELISA substrate (T3405, Sigma-Aldrich, USA) was added, the plates    were developed until the top dilution reached the saturation pointes, and the reaction was stopped with H2SO4. Plates were read at an absorbance of 450 nm.

## Statistical Methods

### Treatment of Missing Data

In the subset of individuals in the returning student subgroup that had ELISA results, there are few missing values for the model variables, with the exception of “working as a service professional” (392/625). As a result, service professional was removed as a predictor in the model. Exploration of these missing values demonstrate no bias by outcome (Figure S1, Figure S2, Table S1, Table S2), confirmed using Chi-squared tests of missingness in predictors by outcome level. Little’s test of Missing Completely At Random (MCAR) indicated that the data was not MCAR (p = 0.048)^2^, and multiple imputation methods (MICE, k-Nearest Neighbour with 5 neighbours, and Bagged Tree)^3–5^ were used to compare model fits (Figure S3). Most missing values occurred across all variables (Figure S2), and there was no observable pattern among the majority of variables: there was some evidence that missingness in “travel in the 3 months prior to return” was associated with “travelling since campus return” response, and that missingness in “eaten in a restaurant in the past 7 days” was associated with “IgG classification”. As such, the predictor variables were deemed to be ‘Missing At Random’, and MICE was used to impute missing values.

### Alternative Estimate of True Prevalence

In the main text we present estimates of the true prevalence in the returning student and community resident cohorts that corrects for the sensitivity of the assay. We estimated sensitivity based on the returning student samples only because the student population had high access to RT-PCR diagnostic tests. Here we present an alternative analysis using an estimate of sensitivity including the community residents. 52 community residents self-reported a positive COVID-19 diagnostic test prior to the first visit; an additional 7 community residents reported a positive COVID-19 diagnostic test between the first and second visit. Of these 7 were positive for IgG antibodies. Pooled with the student results, this results in a sensitivity estimate of 0.611 (95% CI: 0.528 – 0.689). This implies a lower sensitivity in the community resident participants, though the number of observations is low. Figure S4 shows the estimated true prevalence assuming a uniform prior on the interval (0.528, 0.689) on sensitivity in the community resident population and a uniform prior on the interval (0.85, 0.99). For all values of specificity greater than 0.85, there is no change in the qualitative result that the 95% confidence intervals for prevalence in the community residents overlap for both visits and are distinctly different to the prevalence within the returning student subgroup.

## References

1. 	Gontu A, Srinivasan S, Nair MS, et al. Quantitative Estimation of IgM and IgG Antibodies Against SARS-CoV-2. *protocols.io*. Published online August 20, 2020. doi:dx.doi.org/10.17504/protocols.io.bivgke3w

2. 	Little RJA. A Test of Missing Completely at Random for Multivariate Data with Missing Values. *J Am Stat Assoc*. 1988;83(404):1198-1202. doi:10.1080/01621459.1988.10478722

3. 	Kuhn M, Wickham H. *Tidymodels: A Collection of Packages for Modeling and Machine Learning Using Tidyverse Principles*.; 2020. https://www.tidymodels.org

4. 	Buuren S van, Groothuis-Oudshoorn K. mice: Multivariate Imputation by Chained Equations in R. *J Stat Softw*. 2011;45(1):1-67. doi:10.18637/jss.v045.i03

5. 	Kuhn M, Johnson K. *Feature Engineering and Selection: A Practical Approach for Predictive Models*. Taylor & Francis; 2019. Accessed January 30, 2021. https://bookdown.org/max/FES/

## Tables

### Table S1

A comparison of missing data in the subset of individuals with PSU assay results, broken down by exposures vs outcome. P-value refers to Chi-square test with Yates’ continuity correction of missing vs not missing in the predictor variable by outcome level.

| **Variable/Response** | **Negative (N=430)** | **Positive (N=195)** | **Overall (N=625)** | **p-value** |
| --- | --- | --- | --- | --- |
| Close proximity to known COVID-19 Positive Individual |  |  |  | 1 |
| No | 259 (60.2%) | 57 (29.2%) | 316 (50.6%) |  |
| Yes | 170 (39.5%) | 137 (70.3%) | 307 (49.1%) |  |
| Missing | 1 (0.2%) | 1 (0.5%) | 2 (0.3%) |  |
| Attended a gathering of 20-50 people since arrival for the Fall Semester |  |  |  | 1 |
| No | 257 (59.8%) | 75 (38.5%) | 332 (53.1%) |  |
| Yes | 171 (39.8%) | 119 (61.0%) | 290 (46.4%) |  |
| Missing | 2 (0.5%) | 1 (0.5%) | 3 (0.5%) |  |
| Ate in a dining hall in the past 7 days |  |  |  | 0.785 |
| No | 350 (81.4%) | 150 (76.9%) | 500 (80.0%) |  |
| Yes | 78 (18.1%) | 43 (22.1%) | 121 (19.4%) |  |
| Missing | 2 (0.5%) | 2 (1.0%) | 4 (0.6%) |  |
| Attended a gathering of 51-200 people since arrival for the Fall Semester |  |  |  | 0.998 |
| No | 364 (84.7%) | 146 (74.9%) | 510 (81.6%) |  |
| Yes | 61 (14.2%) | 46 (23.6%) | 107 (17.1%) |  |
| Missing | 5 (1.2%) | 3 (1.5%) | 8 (1.3%) |  |
| Travelled in the 3 months prior to campus arrival |  |  |  | 1 |
| No | 189 (44.0%) | 74 (37.9%) | 263 (42.1%) |  |
| Yes | 225 (52.3%) | 114 (58.5%) | 339 (54.2%) |  |
| Missing | 16 (3.7%) | 7 (3.6%) | 23 (3.7%) |  |
| Ate in a restaurant in the past 7 days |  |  |  | 0.051 |
| No | 227 (52.8%) | 90 (46.2%) | 317 (50.7%) |  |
| Yes | 203 (47.2%) | 102 (52.3%) | 305 (48.8%) |  |
| Missing | 0 (0%) | 3 (1.5%) | 3 (0.5%) |  |
| Only ate in their room in the past 7 days |  |  |  | 0.181 |
| No | 144 (33.5%) | 70 (35.9%) | 214 (34.2%) |  |
| Yes | 286 (66.5%) | 123 (63.1%) | 409 (65.4%) |  |
| Missing | 0 (0%) | 2 (1.0%) | 2 (0.3%) |  |
| Close proximity to individual showing COVID-19 symptoms |  |  |  | 1 |
| No | 321 (74.7%) | 121 (62.1%) | 442 (70.7%) |  |
| Yes | 107 (24.9%) | 73 (37.4%) | 180 (28.8%) |  |
| Missing | 2 (0.5%) | 1 (0.5%) | 3 (0.5%) |  |
| Travelled since campus arrival |  |  |  | 1 |
| No | 175 (40.7%) | 80 (41.0%) | 255 (40.8%) |  |
| Yes | 254 (59.1%) | 114 (58.5%) | 368 (58.9%) |  |
| Missing | 1 (0.2%) | 1 (0.5%) | 2 (0.3%) |  |
| Lives in University housing |  |  |  | 1 |
| No | 305 (70.9%) | 140 (71.8%) | 445 (71.2%) |  |
| Yes | 124 (28.8%) | 54 (27.7%) | 178 (28.5%) |  |
| Missing | 1 (0.2%) | 1 (0.5%) | 2 (0.3%) |  |

### Table S2

A comparison of missing data in the subset of individuals with PSU assay results. The variable in bold refers to the variable with missingness being evaluated, and the other variables refer to the association being evaluated. P-value refers to Chi-square test with Yates’ continuity correction of missing vs not missing in the outcome variable by predictor level

| **Variables (Missing vs other)** | **Not missing, n (%)** | **Missing, n (%)** | **p-value** |
| --- | --- | --- | --- |
| **Missing data analysis: Close proximity to known COVID-19 Positive Individual** |  |  |  |
| IgG Classification |  |  |  |
| Negative | 429 (99.8) | 1 (0.2) | 1 |
| Positive | 194 (99.5) | 1 (0.5) |  |
| Travelled in the 3 months prior to campus arrival |  |  |  |
| No | 263 (100.0) | 0 (0.0) | 1 |
| Yes | 338 (99.7) | 1 (0.3) |  |
| Ate in a dining hall in the past 7 days |  |  |  |
| No | 499 (99.8) | 1 (0.2) | 1 |
| Yes | 121 (100.0) | 0 (0.0) |  |
| Ate in a restaurant in the past 7 days |  |  |  |
| No | 317 (100.0) | 0 (0.0) | 0.985 |
| Yes | 304 (99.7) | 1 (0.3) |  |
| Only ate in your room in the past 7 days |  |  |  |
| No | 213 (99.5) | 1 (0.5) | 0.742 |
| Yes | 409 (100.0) | 0 (0.0) |  |
| Lives in University housing |  |  |  |
| No | 444 (99.8) | 1 (0.2) | 1 |
| Yes | 178 (100.0) | 0 (0.0) |  |
| **Missing data analysis: Close proximity to individual showing COVID-19 symptoms** |  |  |  |
| IgG Classification |  |  |  |
| Negative | 428 (99.5) | 2 (0.5) | 1 |
| Positive | 194 (99.5) | 1 (0.5) |  |
| Close proximity to known COVID-19 Positive Individual |  |  |  |
| No | 315 (99.7) | 1 (0.3) | 1 |
| Yes | 307 (100.0) | 0 (0.0) |  |
| Travelled in the 3 months prior to campus arrival |  |  |  |
| No | 263 (100.0) | 0 (0.0) | 0.594 |
| Yes | 337 (99.4) | 2 (0.6) |  |
| Travelled since campus arrival |  |  |  |
| No | 255 (100.0) | 0 (0.0) | 1 |
| Yes | 367 (99.7) | 1 (0.3) |  |
| Attended a gathering of 20-50 people since arrival for the Fall Term |  |  |  |
| No | 331 (99.7) | 1 (0.3) | 1 |
| Yes | 290 (100.0) | 0 (0.0) |  |
| Attended a gathering of 51-200 people since arrival for the Fall Term |  |  |  |
| No | 509 (99.8) | 1 (0.2) | 1 |
| Yes | 107 (100.0) | 0 (0.0) |  |
| Eaten in a dining hall in the past 7 days |  |  |  |
| No | 498 (99.6) | 2 (0.4) | 1 |
| Yes | 121 (100.0) | 0 (0.0) |  |
| Eaten in a restaurant in the past 7 days |  |  |  |
| No | 316 (99.7) | 1 (0.3) | 1 |
| Yes | 304 (99.7) | 1 (0.3) |  |
| Only eaten in your room in the past 7 days |  |  |  |
| No | 213 (99.5) | 1 (0.5) | 1 |
| Yes | 408 (99.8) | 1 (0.2) |  |
| Lives in University housing |  |  |  |
| No | 443 (99.6) | 2 (0.4) | 0.911 |
| Yes | 178 (100.0) | 0 (0.0) |  |
| **Missing data analysis: Attended a gathering of 20-50 people since arrival for the Fall Semester** |  |  |  |
| IgG Classification |  |  |  |
| Negative | 428 (99.5) | 2 (0.5) | 1 |
| Positive | 194 (99.5) | 1 (0.5) |  |
| Close proximity to known COVID-19 Positive Individual |  |  |  |
| No | 315 (99.7) | 1 (0.3) | 1 |
| Yes | 307 (100.0) | 0 (0.0) |  |
| Close proximity to individual showing COVID-19 symptoms |  |  |  |
| No | 441 (99.8) | 1 (0.2) | 1 |
| Yes | 180 (100.0) | 0 (0.0) |  |
| Travelled in the 3 months prior to campus arrival |  |  |  |
| No | 262 (99.6) | 1 (0.4) | 1 |
| Yes | 338 (99.7) | 1 (0.3) |  |
| Travelled since campus arrival |  |  |  |
| No | 254 (99.6) | 1 (0.4) | 0.854 |
| Yes | 368 (100.0) | 0 (0.0) |  |
| Attended a gathering of 51-200 people since arrival for the Fall Term |  |  |  |
| No | 509 (99.8) | 1 (0.2) | 1 |
| Yes | 107 (100.0) | 0 (0.0) |  |
| Eaten in a dining hall in the past 7 days |  |  |  |
| No | 499 (99.8) | 1 (0.2) | 0.844 |
| Yes | 120 (99.2) | 1 (0.8) |  |
| Eaten in a restaurant in the past 7 days |  |  |  |
| No | 317 (100.0) | 0 (0.0) | 0.462 |
| Yes | 303 (99.3) | 2 (0.7) |  |
| Only eaten in your room in the past 7 days |  |  |  |
| No | 212 (99.1) | 2 (0.9) | 0.225 |
| Yes | 409 (100.0) | 0 (0.0) |  |
| Lives in University housing |  |  |  |
| No | 443 (99.6) | 2 (0.4) | 0.911 |
| Yes | 178 (100.0) | 0 (0.0) |  |
| **Missing data analysis: Attended a gathering of 51-200 people since arrival for the Fall Semester** |  |  |  |
| IgG Classification |  |  |  |
| Negative | 425 (98.8) | 5 (1.2) | 0.998 |
| Positive | 192 (98.5) | 3 (1.5) |  |
| Close proximity to known COVID-19 Positive Individual |  |  |  |
| No | 314 (99.4) | 2 (0.6) | 0.656 |
| Yes | 303 (98.7) | 4 (1.3) |  |
| Close proximity to individual showing COVID-19 symptoms |  |  |  |
| No | 439 (99.3) | 3 (0.7) | 0.49 |
| Yes | 177 (98.3) | 3 (1.7) |  |
| Travelled in the 3 months prior to campus arrival |  |  |  |
| No | 260 (98.9) | 3 (1.1) | 1 |
| Yes | 335 (98.8) | 4 (1.2) |  |
| Travelled since campus arrival |  |  |  |
| No | 250 (98.0) | 5 (2.0) | 0.088 |
| Yes | 367 (99.7) | 1 (0.3) |  |
| Attended a gathering of 20-50 people since arrival for the Fall Term |  |  |  |
| No | 330 (99.4) | 2 (0.6) | 0.563 |
| Yes | 286 (98.6) | 4 (1.4) |  |
| Eaten in a dining hall in the past 7 days |  |  |  |
| No | 495 (99.0) | 5 (1.0) | 0.896 |
| Yes | 119 (98.3) | 2 (1.7) |  |
| Eaten in a restaurant in the past 7 days |  |  |  |
| No | 315 (99.4) | 2 (0.6) | 0.417 |
| Yes | 300 (98.4) | 5 (1.6) |  |
| Only eaten in your room in the past 7 days |  |  |  |
| No | 211 (98.6) | 3 (1.4) | 0.939 |
| Yes | 405 (99.0) | 4 (1.0) |  |
| Lives in University housing |  |  |  |
| No | 440 (98.9) | 5 (1.1) | 0.846 |
| Yes | 177 (99.4) | 1 (0.6) |  |
| **Missing data analysis: Lives in University housing** |  |  |  |
| IgG Classification |  |  |  |
| Negative | 429 (99.8) | 1 (0.2) | 1 |
| Positive | 194 (99.5) | 1 (0.5) |  |
| Close proximity to known COVID-19 Positive Individual |  |  |  |
| No | 315 (99.7) | 1 (0.3) | 1 |
| Yes | 307 (100.0) | 0 (0.0) |  |
| Close proximity to individual showing COVID-19 symptoms |  |  |  |
| No | 441 (99.8) | 1 (0.2) | 1 |
| Yes | 180 (100.0) | 0 (0.0) |  |
| Travelled in the 3 months prior to campus arrival |  |  |  |
| No | 262 (99.6) | 1 (0.4) | 1 |
| Yes | 338 (99.7) | 1 (0.3) |  |
| Travelled since campus arrival |  |  |  |
| No | 255 (100.0) | 0 (0.0) | 1 |
| Yes | 367 (99.7) | 1 (0.3) |  |
| Attended a gathering of 20-50 people since arrival for the Fall Term |  |  |  |
| No | 332 (100.0) | 0 (0.0) | 0.946 |
| Yes | 289 (99.7) | 1 (0.3) |  |
| Eaten in a dining hall in the past 7 days |  |  |  |
| No | 499 (99.8) | 1 (0.2) | 0.844 |
| Yes | 120 (99.2) | 1 (0.8) |  |
| Eaten in a restaurant in the past 7 days |  |  |  |
| No | 317 (100.0) | 0 (0.0) | 0.462 |
| Yes | 303 (99.3) | 2 (0.7) |  |
| Only eaten in your room in the past 7 days |  |  |  |
| No | 212 (99.1) | 2 (0.9) | 0.225 |
| Yes | 409 (100.0) | 0 (0.0) |  |
| **Missing data analysis: Travelled in the 3 months prior to campus arrival** |  |  |  |
| IgG Classification |  |  |  |
| Negative | 414 (96.3) | 16 (3.7) | 1 |
| Positive | 188 (96.4) | 7 (3.6) |  |
| Close proximity to known COVID-19 Positive Individual |  |  |  |
| No | 309 (97.8) | 7 (2.2) | 0.112 |
| Yes | 292 (95.1) | 15 (4.9) |  |
| Close proximity to individual showing COVID-19 symptoms |  |  |  |
| No | 429 (97.1) | 13 (2.9) | 0.307 |
| Yes | 171 (95.0) | 9 (5.0) |  |
| Travelled since campus arrival |  |  |  |
| No | 252 (98.8) | 3 (1.2) | 0.015 |
| Yes | 349 (94.8) | 19 (5.2) |  |
| Attended a gathering of 20-50 people since arrival for the Fall Term |  |  |  |
| No | 324 (97.6) | 8 (2.4) | 0.158 |
| Yes | 276 (95.2) | 14 (4.8) |  |
| Attended a gathering of 51-200 people since arrival for the Fall Term |  |  |  |
| No | 495 (97.1) | 15 (2.9) | 0.124 |
| Yes | 100 (93.5) | 7 (6.5) |  |
| Eaten in a dining hall in the past 7 days |  |  |  |
| No | 482 (96.4) | 18 (3.6) | 1 |
| Yes | 117 (96.7) | 4 (3.3) |  |
| Eaten in a restaurant in the past 7 days |  |  |  |
| No | 306 (96.5) | 11 (3.5) | 1 |
| Yes | 294 (96.4) | 11 (3.6) |  |
| Only eaten in your room in the past 7 days |  |  |  |
| No | 207 (96.7) | 7 (3.3) | 0.979 |
| Yes | 394 (96.3) | 15 (3.7) |  |
| Lives in University housing |  |  |  |
| No | 426 (95.7) | 19 (4.3) | 0.33 |
| Yes | 174 (97.8) | 4 (2.2) |  |
| **Missing data analysis: Travelled since campus arrival** |  |  |  |
| IgG Classification |  |  |  |
| Negative | 429 (99.8) | 1 (0.2) | 1 |
| Positive | 194 (99.5) | 1 (0.5) |  |
| Travelled in the 3 months prior to campus arrival |  |  |  |
| No | 263 (100.0) | 0 (0.0) | 1 |
| Yes | 338 (99.7) | 1 (0.3) |  |
| Eaten in a dining hall in the past 7 days |  |  |  |
| No | 499 (99.8) | 1 (0.2) | 1 |
| Yes | 121 (100.0) | 0 (0.0) |  |
| Eaten in a restaurant in the past 7 days |  |  |  |
| No | 317 (100.0) | 0 (0.0) | 0.985 |
| Yes | 304 (99.7) | 1 (0.3) |  |
| Only eaten in your room in the past 7 days |  |  |  |
| No | 213 (99.5) | 1 (0.5) | 0.742 |
| Yes | 409 (100.0) | 0 (0.0) |  |
| Lives in University housing |  |  |  |
| No | 444 (99.8) | 1 (0.2) | 1 |
| Yes | 178 (100.0) | 0 (0.0) |  |
| **Missing data analysis: Only eaten in your room in the past 7 days** |  |  |  |
| IgG Classification |  |  |  |
| Negative | 430 (100.0) | 0 (0.0) | 0.181 |
| Positive | 193 (99.0) | 2 (1.0) |  |
| Close proximity to known COVID-19 Positive Individual |  |  |  |
| No | 316 (100.0) | 0 (0.0) | 0.988 |
| Yes | 306 (99.7) | 1 (0.3) |  |
| Close proximity to individual showing COVID-19 symptoms |  |  |  |
| No | 442 (100.0) | 0 (0.0) | 0.642 |
| Yes | 179 (99.4) | 1 (0.6) |  |
| Travelled in the 3 months prior to campus arrival |  |  |  |
| No | 262 (99.6) | 1 (0.4) | 0.899 |
| Yes | 339 (100.0) | 0 (0.0) |  |
| Travelled since campus arrival |  |  |  |
| No | 255 (100.0) | 0 (0.0) | 1 |
| Yes | 367 (99.7) | 1 (0.3) |  |
| Attended a gathering of 20-50 people since arrival for the Fall Term |  |  |  |
| No | 331 (99.7) | 1 (0.3) | 1 |
| Yes | 290 (100.0) | 0 (0.0) |  |
| Attended a gathering of 51-200 people since arrival for the Fall Term |  |  |  |
| No | 509 (99.8) | 1 (0.2) | 1 |
| Yes | 107 (100.0) | 0 (0.0) |  |
| Lives in University housing |  |  |  |
| No | 444 (99.8) | 1 (0.2) | 1 |
| Yes | 177 (99.4) | 1 (0.6) |  |
| **Missing data analysis: Eaten in a restaurant in the past 7 days** |  |  |  |
| IgG Classification |  |  |  |
| Negative | 430 (100.0) | 0 (0.0) | 0.051 |
| Positive | 192 (98.5) | 3 (1.5) |  |
| Close proximity to known COVID-19 Positive Individual |  |  |  |
| No | 316 (100.0) | 0 (0.0) | 0.466 |
| Yes | 305 (99.3) | 2 (0.7) |  |
| Close proximity to individual showing COVID-19 symptoms |  |  |  |
| No | 441 (99.8) | 1 (0.2) | 1 |
| Yes | 179 (99.4) | 1 (0.6) |  |
| Travelled in the 3 months prior to campus arrival |  |  |  |
| No | 261 (99.2) | 2 (0.8) | 0.371 |
| Yes | 339 (100.0) | 0 (0.0) |  |
| Travelled since campus arrival |  |  |  |
| No | 254 (99.6) | 1 (0.4) | 1 |
| Yes | 367 (99.7) | 1 (0.3) |  |
| Attended a gathering of 20-50 people since arrival for the Fall Term |  |  |  |
| No | 331 (99.7) | 1 (0.3) | 1 |
| Yes | 289 (99.7) | 1 (0.3) |  |
| Attended a gathering of 51-200 people since arrival for the Fall Term |  |  |  |
| No | 508 (99.6) | 2 (0.4) | 1 |
| Yes | 107 (100.0) | 0 (0.0) |  |
| Eaten in a dining hall in the past 7 days |  |  |  |
| No | 500 (100.0) | 0 (0.0) | 0.441 |
| Yes | 120 (99.2) | 1 (0.8) |  |
| Only eaten in your room in the past 7 days |  |  |  |
| No | 213 (99.5) | 1 (0.5) | 0.742 |
| Yes | 409 (100.0) | 0 (0.0) |  |
| Lives in University housing |  |  |  |
| No | 444 (99.8) | 1 (0.2) | 0.41 |
| Yes | 176 (98.9) | 2 (1.1) |  |
| **Missing data analysis: Eaten in a dining hall in the past 7 days** |  |  |  |
| IgG Classification |  |  |  |
| Negative | 428 (99.5) | 2 (0.5) | 0.785 |
| Positive | 193 (99.0) | 2 (1.0) |  |
| Close proximity to known COVID-19 Positive Individual |  |  |  |
| No | 314 (99.4) | 2 (0.6) | 1 |
| Yes | 306 (99.7) | 1 (0.3) |  |
| Close proximity to individual showing COVID-19 symptoms |  |  |  |
| No | 441 (99.8) | 1 (0.2) | 0.42 |
| Yes | 178 (98.9) | 2 (1.1) |  |
| Travelled in the 3 months prior to campus arrival |  |  |  |
| No | 261 (99.2) | 2 (0.8) | 0.825 |
| Yes | 338 (99.7) | 1 (0.3) |  |
| Travelled since campus arrival |  |  |  |
| No | 253 (99.2) | 2 (0.8) | 0.749 |
| Yes | 367 (99.7) | 1 (0.3) |  |
| Attended a gathering of 20-50 people since arrival for the Fall Term |  |  |  |
| No | 331 (99.7) | 1 (0.3) | 0.906 |
| Yes | 288 (99.3) | 2 (0.7) |  |
| Attended a gathering of 51-200 people since arrival for the Fall Term |  |  |  |
| No | 507 (99.4) | 3 (0.6) | 0.975 |
| Yes | 107 (100.0) | 0 (0.0) |  |
| Eaten in a restaurant in the past 7 days |  |  |  |
| No | 316 (99.7) | 1 (0.3) | 1 |
| Yes | 304 (99.7) | 1 (0.3) |  |
| Only eaten in your room in the past 7 days |  |  |  |
| No | 213 (99.5) | 1 (0.5) | 1 |
| Yes | 408 (99.8) | 1 (0.2) |  |
| Lives in University housing |  |  |  |
| No | 444 (99.8) | 1 (0.2) | 0.132 |
| Yes | 175 (98.3) | 3 (1.7) |  |

### Table S3

IgG ELISA results as a function of self-reported prior SARS-CoV-2 diagnostic test outcome among returning student cohort participants. 

|  | **Prior Test (N=665)** |  | **No Prior Test (N=42)** |
| --- | --- | --- | --- |
| **ELISA Result** | **Prior Positive (N=105)** | **No Prior Positive (N=560)** |  |
| Positive | 84 | 106 | 5 |
| Negative | 6 | 390 | 34 |
| NA | 15 | 64 | 3 |

### Table S4

Propensity of following public health measures in returning students and community members with PSU ELISA results; subset of community members that received the “Health Messaging” survey. P-value refers to Chi-square test with Yates’ continuity correction of proportions in the predictor level by cohort.

|  | **Returning Students (N=625)** | **Community – Health Messaging (N=242)** | **p-value** |
| --- | --- | --- | --- |
| Mask Wearing, n (%) |  |  | < 0.001 |
| Not Always | 83 (13.3) | 56 (23.1) |  |
| Always | 540 (86.4) | 186 (76.9) |  |
| Missing | 2 (0.3) | 0 (0) |  |
| Distancing in Public, n (%) |  |  | 0.584 |
| Not Always | 442 (70.7) | 166 (68.6) |  |
| Always | 180 (28.8) | 75 (31.0) |  |
| Missing | 3 (0.5) | 1 (0.4) |  |
| Avoiding crowds of >25 people, n (%) |  |  | < 0.001 |
| Not Always | 355 (56.8) | 83 (34.3) |  |
| Always | 268 (42.9) | 159 (65.7) |  |
| Missing | 2 (0.3) | 0 (0) |  |

### Table S5

Raw prevalence in each subgroup by adherence to PH public health measures

| **Variable/Response** | **Students (N=625)** |  | **Community (N=242)** |  |
| --- | --- | --- | --- | --- |
|  | **Negative** | **Positive** | **Negative** | **Positive** |
| Mask Wearing, n (%) |  |  |  |  |
| Not Always | 59 (71.1) | 24 (28.9) | 52 (92.9) | 4 (7.1) |
| Always | 370 (68.5) | 170 (31.5) | 168 (90.3) | 18 (9.7) |
| Missing | 1 (50.0) | 1 (50.0) | 0 (0.0) | 0 (0.0) |
| Distancing in Public, n (%) |  |  |  |  |
| Not Always | 292 (66.1) | 150 (33.9) | 152 (91.6) | 14 (8.4) |
| Always | 136 (75.6) | 44 (24.4) | 67 (89.3) | 8 (10.7) |
| Missing | 2 (66.7) | 1 (33.3) | 0 (0.0) | 0 (0.0) |
| Avoiding crowds of >25 people, n (%) |  |  |  |  |
| Not Always | 230 (64.8) | 125 (35.2) | 74 (89.2) | 9 (10.8) |
| Always | 199 (74.3) | 69 (25.7) | 146 (91.8) | 13 (8.2) |
| Missing | 1 (50.0) | 1 (50.0) | 0 (0.0) | 0 (0.0) |

## Figures

### Figure S1

Co-occurrence plot of missing predictor values in subset of returning students with PSU ELISA

### Figure S2

Comparison of proportions missing for all model variables

### Figure S3

Multiple imputation method effects. 

### Figure S4

Sensitivity analysis of true prevalence amongst returning student and community subgroups, using pooled estimate of IgG test sensitivity against self-reported prior positive test
